# Differential gene expression between *in vivo* and *in vitro* maturation: a comparative study with bovine oocytes derived from the same donor pool

**DOI:** 10.5935/1518-0557.20180084

**Published:** 2019

**Authors:** Luiz Sergio Almeida Camargo, Michele Munk, Jose Nelio Sales, Sabine Wohlres-Viana, Carolina Capobiango Romano Quintão, João Henrique Moreira Viana

**Affiliations:** 1 Brazilian Agricultural Research Corporation (Embrapa) - Dairy Cattle, Juiz de Fora, MG, Brazil; 2 Federal University of Juiz de Fora, Juiz de Fora, MG, Brazil; 3 Federal University of Lavras, Lavras, MG, Brazil; 4 Embrapa - Cenargen, Brasilia, DF, Brazil

**Keywords:** *in vivo* maturation, mRNA, gene expression, ovum pick-up

## Abstract

**Objective:**

*In vitro* maturation has been shown to influence gene expression in oocytes, but a common shortcoming in reports on the matter has been the use of different donors in each experimental group thus disregarding donor effects. This study aimed to investigate the abundance of mRNA in oocytes matured *in vivo* and *in vitro* obtained from the same group of donors.

**Methods:**

A bovine model was used to assess the relative abundance of specific transcripts in in vitro-matured (IN VITRO-OPU) and *in vivo*-matured (IN VIVO-OPU) oocytes collected from the same donors by transvaginal ovum pick-up (OPU). Transcript abundance in oocytes from the IN VIVO-OPU group and oocytes matured *in vitro* but retrieved from different cows slaughtered at a commercial abattoir (IN VITRO-Abattoir group) was also compared. Total RNA was extracted from denuded oocytes and cDNA was produced via reverse transcription using an oligo(dT) primer for relative quantification of eight target transcripts by real-time PCR.

**Results:**

Oocytes in the IN VITRO-OPU group had lower (*p*<0.05) abundance of peroxiredoxin 1 (*Prdx1*), heat shock protein 70.1 (*Hsp70.1*), growth and differentiation factor 9 (*Gdf9*), and maternal antigen that embryo requires (*Mater*) transcripts than the oocytes in the IN VIVO-OPU group, all obtained from the same pool of donor cows. Similar results were seen in the comparisons involving the IN VIVO-OPU and IN VITRO-Abattoir groups (*p*<0.05).

**Conclusion:**

*In vitro* maturation affected the abundance of polyadenylated transcripts in the oocyte cytoplasm when compared to *in vivo* maturation induced by exogenous hormones in oocytes collected from the same donor pool.

## INTRODUCTION

In the roster of assisted reproductive technologies (ART), oocyte *in vitro* maturation (IVM) can be an alternative for patients with ovarian conditions prescribed treatments that may compromise their oocytes (Reinblatt & Buckett, 2008; Ata *et al*., 2010; Shalom-Paz *et al*., 2010) and individuals at risk of ovarian hyperstimulation syndrome (Sauerbrun-Cutler *et al*., 2015). IVM may also decrease the number of clinical consultations and the level to which patients may require drug therapy, thus reducing the cost of treatment (Picton, 2002). Despite its potential benefits, IVM is still marginally used in human ART, with applications mainly in fertility preservation (Kasum *et al*., 2015; Lambertini *et al*., 2015, Shirasawa & Terada, 2017). IVM is limited by oocyte developmental competence (Gilchrist & Thompson, 2007) and may result in lower fertilization and embryo production rates (Banwell & Thompson, 2008). IVM may also disturb the meiotic spindle, the morphology of human oocyte chromosomal alignment (Li *et al*., 2006), and expose gametes to reactive oxygen species (Combelles *et al*., 2009). Lower implantation and birth rates have been reported with IVM, when compared to conventional protocols in which maturation is induced *in vivo* (Suikkari, 2008; Smitz *et al*., 2011). Concerns over the long-term effects of IVM on offspring health have been reported (Suikkari & Soderstrom-Anttila, 2007). Unfortunately, little is known about oocyte maturation when compared to other developmental processes (Coticchio *et al*., 2015). Therefore, a better understanding of the mechanisms involved in oocyte competence acquisition and the factors that might disturb it during IVM is crucial in the optimization of this technology.

During IVM, oocytes need to undergo nuclear and cytoplasmic maturation. Meiosis resumes and progresses up to metaphase II, while the cytoplasmic organelles undergo redistribution (Ferreira *et al*., 2009) required for cortical granule secretion and pronuclear formation. Nevertheless, before becoming fully competent oocytes also need to go through molecular maturation. Transcriptional activity is supposed to be low during maturation (Bettegowda & Smith, 2007) and much of the mRNA transcribed and stored in the cytoplasm during oocyte growth is degraded, whereas some of it is protected from degradation and conferred stability by the 3' untranslated regions (Brevini *et al*., 2007). Polyadenylated oocyte mRNAs are required not only for meiotic resumption, but also for early embryo development (Piccioni *et al*., 2005; Brevini *et al*., 2007; Evsikov & Marín de Evsikova, 2009). Studies have shown that during oocyte maturation some mRNA can undergo deadenylation (Brevini-Gandolfi *et al*., 1999; Lequarre *et al*., 2004), while other transcripts may accumulate in polyadenylated form (Tomek *et al*., 2002). A recent study using single oocytes and RNA-Seq showed that some polyadenylated transcripts increase while others decrease in abundance, showing the importance of cytoplasmic polyadenylation during oocyte maturation (Reyes *et al*., 2015). Different forms of mRNA and proteins are present in the ooplasm and may be required in early cleavage (Meirelles *et al*., 2004; Li *et al*., 2010), playing a role on embryonic genome activation (Schultz, 2002).

Previous studies reported that IVM might impact oocyte gene expression and alter the amount of mRNA stored in the ooplasm, which in turn would affect further embryo development. However, there is no consensus over such effect of IVM. A study showed that Rhesus monkey oocytes matured *in vitro* had different levels of expression of some maternal mRNAs when compared to oocytes matured *in vivo* (Zheng *et al*., 2005), while other authors described close similarities between oocytes matured *in vitro* and *in vivo* (Lee *et al*., 2008). The authors of the latter study performed cDNA-array analysis and found only 59 genes differentially expressed between oocytes matured *in vitro* and *in vivo*, which accounted for a mere 0.31% of the total probe set analyzed. In humans, global gene expression analysis revealed that more than 2,000 genes were differentially expressed between oocytes matured *in vivo* and *in vitro* (Jones *et al*., 2008). Despite a few differences, another study found that human oocytes matured *in vivo* and *in vitro* shared similar patterns of gene expression (Wells & Patrizio, 2008). In bovines, IVM impacted the amount of mRNA transcripts stored in the ooplasm when compared to *in vivo* maturation (Lonergan *et al*., 2003). Transcriptome analysis found distinct transcription patterns between bovine oocytes matured *in vivo* and *in vitro* (Katz-Jaffe *et al*., 2009). Therefore, the impact of IVM on oocyte transcriptome is unclear. A downside common to these studies is the use of different donors in each experimental group. Since oocytes accumulate transcripts as they grow, differences among donors in the amount of mRNA stored in the oocytes before maturation might affect the interpretation of the effects of IVM on the abundance of transcripts in the ooplasm.

This study aimed to investigate the abundance of polyadenylated mRNA in oocyte pools matured *in vivo* and *in vitro* harvested from the same group of cows in order to decrease the effects of individual variation among donors in the amount of transcripts. The study also compared the abundance of polyadenylated mRNA between oocytes matured *in vivo* and oocytes matured *in vitro* retrieved from different cows in order to evaluate whether the effects of IVM on mRNA abundance might also be seen in oocytes coming from different donors. A bovine model was used because of the practical and ethical limitations inherent to working with human oocytes, the similarities between human and bovine oocyte maturation (Ménézo & Hérubel, 2002), and the well-established *in vitro* maturation protocols for bovine oocytes. The following genes were chosen according to the importance of their proteins for oocyte and early embryo development: maternal antigen that embryo requires (*Mater*), zygote arrest 1 (*Zar1*), growth and differentiation factor 9 (*Gdf9*), B-cell CLL/lymphoma 2 (*Bcl-2*), BCL-2-associated X protein (*Bax*), peroxiredoxin 1 (*Prdx1*), heat shock protein 70.1 (*Hsp70.1*) and high-mobility-group 1 (*Hmgn1*).

## MATERIALS AND METHODS

### Chemicals and animals 

The chemicals used in this study were purchased from Sigma Chemical (St. Louis, MO, USA) unless otherwise indicated. The oocyte donors were mature crossbred cows kept in pasture with water *ad libitum* and under shadow.

### Experimental design

This study compared the relative abundance of specific transcripts in oocytes obtained by transvaginal ovum pick-up (OPU) of the same donors matured either *in vitro* (IN VITRO-OPU group) or *in vivo* (IN VIVO-OPU group). Transcript abundance was also compared between oocytes matured *in vivo* (IN VIVO-OPU group) and oocytes matured *in vitro* (IN VITRO-Abattoir group), with the latter obtained from ovaries collected at a commercial abattoir from different cows. Five cows underwent OPU to collect oocytes for *in vitro* maturation (IN VITRO-OPU). Two weeks later, the same donors were submitted to hormone therapy to induce *in vivo* maturation and had their oocytes retrieved by OPU (IN VIVO-OPU group). In the IN VITRO-Abattoir group, the ovaries were retrieved post-mortem at a local commercial abattoir and transported to the laboratory. Maturation was deemed complete when the oocytes had fully expanded cumulus cells. Three pools with 10 matured denuded oocytes in each group were submitted to RNA extraction. Relative quantification of *Prdx1, Hsp70.1, Gdf9, Mater, Zar1, Bax, BCL2*, and *Hmgn1* genes was performed with real-time PCR.

### Ovum Pick-up

The oocytes in the IN VITRO-OPU and IN VIVO-OPU groups were harvested with the aid of a portable ultrasound device equipped with a sector scanner and a 7.5 MHz transvaginal transducer (Aquila Vet, Esaote, Geneva, Italy). Ovum pick-up was performed with disposable 20 gauge needles (WTA Tecnologia, Cravinhos, SP, Brazil) at a vacuum pressure of 80 mmHg. The aspirated follicular fluid was collected in 50 mL tubes containing TALP-HEPES added with 125 IU/ml of heparin (Liquemine, Roche Lab, Brazil). In the IN VITRO-OPU group, the oocytes were harvested from crossbreds between Gir and Holstein not submitted to hormone therapy on a random day of the estrous cycle. In the IN VIVO-OPU group, OPU was performed two weeks later in the same cows. In both IN VIVO- and IN VITRO-OPU groups, all follicles measuring 3-8 mm in diameter were aspirated. The mean ± SEM number of oocytes collected by OPU/donor was 13.7±3.9 and 16.2±3.2 in the IN VIVO- and IN VITRO-OPU groups, respectively.

### Oocytes collected from abattoir ovaries 

The immature oocytes included in the IN VITRO-Abattoir group were aspirated from the follicles of ovaries picked randomly at a local abattoir. The ovaries were transported to the laboratory in 0.9% sodium chloride solution supplemented with 0.05 g/L of streptomycin at 33-37ºC within 3 h. In the laboratory, the ovaries were rinsed in sodium chloride solution at 35-37ºC and follicles measuring 3-8 mm in diameter were aspirated with a 21 G needle attached to a disposable syringe.

### *In vivo* maturation

The oocytes in the IN VIVO-OPU group were harvested after hormonal stimulation of the pre-ovulatory LH surge to induce *in vivo* maturation. The cows were implanted a progesterone-releasing intravaginal device - a controlled internal drug release (CIDR) insert (Pfizer, São Paulo, Brazil) - and prescribed 2 mg of estradiol benzoate (Estrogin, Farmavet, São Paulo, Brazil) on Day 0. On Day 4, the cows were stimulated with 180 mg FSH (Folltropin, Bioniche, Canada) injected in six decreasing doses every 12 h. On Day 6, the cows were administered 0.53 mg of cloprostenol sodium (Ciosin, Cooper, São Paulo, Brazil). On Day 7, the CIDR insert was removed and the cows were injected 2.5 mg of gonadorelin (Gestran-Plus, Tecnopec, São Paulo, Brazil). OPU was performed 18 h after the gonadorelin injection. Only oocytes with expanded cumulus cells and homogeneous cytoplasm (n=37) were selected for the experiment. The selected oocytes were denuded with 0.1% hyaluronidase, pooled in groups of ten, and rapidly frozen in liquid nitrogen for further RNA extraction.

### *In vitro* maturation

In the IN VITRO-OPU (n=40) and IN VITRO-Abattoir groups (n=40), only immature cumulus-oocyte complexes (COCs) with more than three compact layers of cumulus cells and oocytes with homogeneous cytoplasm were selected and taken to *in vitro* maturation. IVM was performed in tissue culture medium (medium 199; Gibco Life Technologies, Grand Island, NY, USA) supplemented with 20 µg/mL^-1^ FSH (Pluset, Serono, Italy), 0.36mM sodium pyruvate, 10mM sodium bicarbonate, and 50 mg/mL^-1^ streptomycin-penicillin in a humidified atmosphere with 5% CO_2_ in air at 38.5ºC for 24 h. Only oocytes with expanded cumulus cell were denuded, pooled, and stored in the same way of the oocytes matured *in vivo*.

### Total RNA extraction and reverse transcription (RT)

Total RNA was extracted from three pools of 10 oocytes per group with the RNeasy Micro Kit (Qiagen, Hilden, Germany) according to manufacturer instructions and treated with DNase to prevent DNA contamination. Elution was performed with 12 µL of RNase-free water. In order to isolate poly(A)^+^ RNA, the samples (8 µL, equivalent to 6.7 oocytes) were submitted to reverse transcription with the SuperScript III First-Strand Synthesis Supermix (Invitrogen, Carlsbad, CA, USA) kit according to manufacturer instructions using oligo(dT)_20_ primers, dNTP mix, Superscript III RT, RNaseOUT, MgCl_2_, and RT buffer in a final volume of 20 µl (equivalent to 0.33 oocyte/µL). The samples were first incubated at 65ºC for 5 min and then at 50ºC for 50 min. The reaction was terminated at 85ºC for 5 min and the samples were then chilled in ice. After that, RNase H was added to the samples and incubated at 37ºC for 20 min. RNA and cDNA from each pool and group were quantified on a spectrophotometer (Nanodrop 2000, Wilmington, DE, USA) using 1 µL of sample. Samples presenting 260/280 ratios between 1.7 and 2.0 were considered appropriate for expression analysis.

### Relative quantification by real-time polymerase chain reaction (PCR)

Relative quantification was performed in triplicate using real-time polymerase chain reaction (ABI Prism 7300 Sequence Detection Systems, Foster City, CA, USA). The reactions were prepared using a mixture of SYBR Green PCR Master Mix (Applied Biosystems), 0.1µM primers, nuclease-free water, and cDNA. The volume of RT (with cDNA) in the PCR reactions was calculated based on oocyte-equivalents. Due to the restriction of RNA amount in the oocytes, only one housekeeping gene was used as an endogenous reference for relative quantification. The beta-ACTIN (*Actb*) gene was chosen as the endogenous reference since it displayed a low coefficient of variation (2.2%) among the samples in the present experiment. Polymerase chain reaction for the *Bax, Bcl-2, Prdx1* and *Hsp70.1* genes was performed with 1 µL of RT reaction (equivalent to 0.33 oocyte/PCR reaction) whereas for the *Gdf9, Mater, Zar1, Hmgn1* and *Actb* genes, PCR was performed with 0.33 µL of RT reaction (equivalent to 0.1 oocyte/PCR reaction). The cDNA template was denatured at 95ºC for 10 min, followed by 45 cycles at 95ºC for 15s, the gene-specific primer annealing temperature for 30s (Table 1) and elongation at 60ºC for 30s. After each PCR run, melting curve analysis was performed to confirm that a single specific product was generated. Negative controls, comprising the PCR reaction mixture without nucleic acids, were also run with each group of samples. Primer efficiency was calculated using the LinRegPCR software (Ramakers *et al*., 2003) for each reaction. The mean primer efficiency was 1.84, 1.63, 1.80, 1.84, 1.81, 1.87, 1.88, 1.85, and 1.84 for *Actb, Bax, Bcl-2, Prdx1, Hsp70.1, Gdf9, Mater, Zar1,* and *Hmgn1*, respectively.

**Table 1:** Primer sequences used for relative gene expression analysis by real-time polymerase chain reaction

Gene	Primer sequences (5’–3’)	Annealing (ºC)	Fragment size (bp)	GenBank accession no.
*Bax*	Forward:TTTGCTTCAGGGTTTCATCCAGGA	64	174	NM_173894
	Reverse: CAGCTGCGATCATCCTCTGCAG			
*Bcl-2*	Forward: TGGATGACCGAGTACCTGAA	53	120	NM_001166486
	Reverse: CAGCCAGGAGAAATCAAACA			
*Prdx1*	Forward: ATGCCAGATGGTCAGTTCAAG	52	224	NM_174431
	Reverse: CCTTGTTTCTTGGGTGTGTTG			
*Hsp70.1*	Forward: AACAAGATCACCATCACCAACG	59	275	NM_174550
	Reverse: TCCTTCTCCGCCAAGGTGTTG			
*Gdf9*	Forward: GACCCCTAAATCCAACAGAA	53	120	NM_174681
	Reverse: AGCAGATCCACTGATGGAA			
*Mater*	Forward: AATGACGACGCTGTGTTCTG	53	206	NM_001007814
	Reverse: GCGGTTCTCAGGTTCTTCAG			
*Zar1*	Forward: TGCCGAACATGCCAGAAG	53	188	NM_001076203
	Reverse: TCACAGGATAGGCGTTTGC			
*Hmgn1*	Forward: GTGGCCAACCAGGAGACTAA	53	147	NM_001034772
	Reverse: AAACAGGGACCACTGACAGG			
*Actb*	Forward: GACATCCGCAAGGACCTCTA	53	205	NM_173979
	Reverse: ACATCTGCTGGAAGGTGGAC			

### Statistical analysis

The Comparative Ct quantification method on the REST software package (Pfaffl *et al*., 2002) was used to perform relative quantification analysis based on primer efficiency. Data from the IN VIVO-OPU group were used as calibrator and set to one. Analysis was performed by a pair-wise fixed reallocation randomization test. *p*<0.05 was considered significant and the relative expression values were presented as mean values ± SEM.

## RESULTS

**Figure 1:**
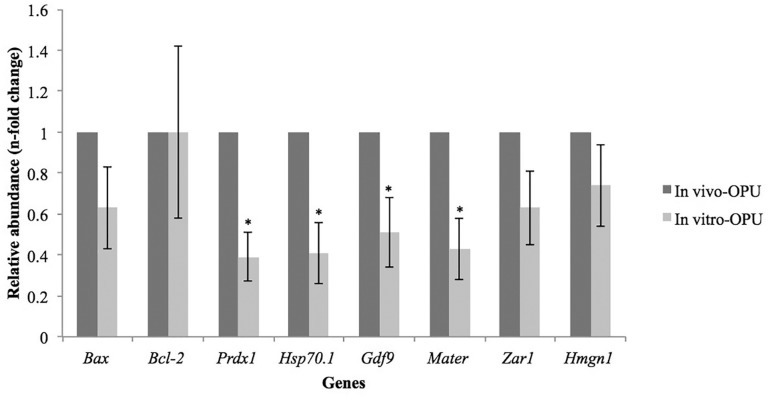
Relative abundance of transcripts from different genes of in vivo-matured (IN VIVO-OPU) and in vitro-matured (IN VITRO-OPU) bovine oocytes derived from the same donors. Transcript level of in vivo-matured oocytes was used as calibrator (relative abundance = 1.00). Data show as mean ± SEM. (*) Asterisk indicates difference between IN VIVO-OPU and IN VITRO-OPU groups (*p*<0.05)

**Figure 2:**
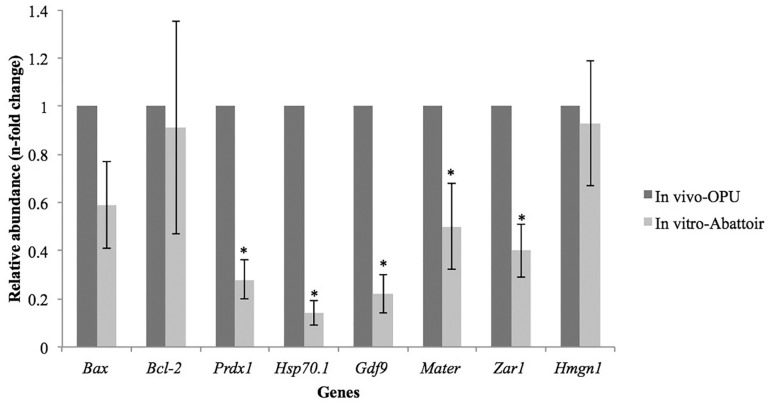
Relative abundance of transcripts from different genes of in vivo-matured (IN VIVO-OPU) and in vitro-matured (IN VITRO-Abattoir) bovine oocytes derived from different donors. Transcript level of in vivo-matured oocytes was used as calibrator (relative abundance = 1.00). Data are show as mean ± SEM. (*) Asterisk indicates difference between IN VIVO-OPU and IN VITRO–Abattoir groups (*p*<0.05)

**Figure 3:**
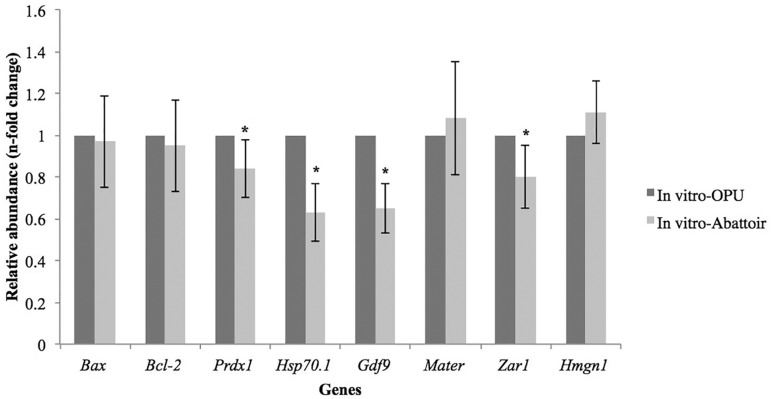
Relative abundance of transcripts from different genes of in vitro-matured bovine oocytes derived from different donors, collected by means of OPU (IN VITRO-OPU) or by post-mortem follicular aspiration (IN VITRO-Abattoir). Transcript level of IN VITRO-OPU oocytes was used as calibrator (relative abundance = 1.00). Data are show as mean ± SEM. (*) Asterisk indicates difference between IN VITRO-OPU and IN VITRO–Abattoir groups (*p*<0.05)

Full cumulus cell expansion was observed at the end of *in vitro* and *in vivo* maturation, but oocytes matured *in vivo* had a more gelatinous matrix around the oocyte. After denudation, only oocytes with a homogenous cytoplasm were used for relative quantification of *Bax, Bcl-2, Prdx1, Hsp70.1, Gdf9, Mater, Zar1*, and *Hmgn1* transcripts. The oocytes collected from the same donors included in the IN VITRO-OPU group had decreased (*p*<0.05) relative abundance of *Prdx1, Hsp70.1, Gdf9*, and *Mater* transcripts when compared to the oocytes in the IN VIVO-OPU group (Figure 1). A similar result was observed when the comparison was performed between oocytes from different cows matured *in vitro* or *in vivo*. The oocytes in the IN VITRO-Abattoir group had decreased abundance of *Prdx1, Hsp70.1, Gdf9*, and *Mater* transcripts than the oocytes in the IN VIVO-OPU group (Figure 2). The abundance of *Zar1* transcripts was also lower in the oocytes in the IN VITRO-Abattoir group (Figure 2). When both *in vitro* maturation groups (IN VITRO-OPU vs. IN VITRO-Abattoir) were compared, lower (*p*<0.05) amounts of *Prdx1, Hsp70.1, Gdf9*, and *Zar1* transcripts were found in the oocytes in the IN VITRO-Abattoir group (Figure 3).

## DISCUSSION

*In vitro* maturation is a critical step to produce developmentally competent oocytes for the *in vitro* production of embryos of domestic species, and may become an important tool in human assisted reproductive technology procedures. However, *in vitro* maturation of mammalian oocytes has been associated with decreased developmental ability, possibly due to cellular and molecular disturbances caused by the *in vitro* environment (Gilchrist & Thompson, 2007; Krisher, 2013). On the other hand, *in vivo* maturation improved the quality and developmental competence of bovine oocytes (Blondin *et al*., 2002; Dieleman *et al*., 2002). However, the effect of IVM on mRNA abundance is controversial (Zheng *et al*., 2005; Jones *et al*., 2008; Lee *et al*., 2008; Wells & Patrizio, 2008), with discrepancies arising from individual oocyte donor-related effects. Differently from other studies, we compared the abundance of polyadenylated transcripts between *in vivo* and *in vitro* matured bovine oocytes obtained from the same donors, and found that IVM affected the relative abundance of specific transcripts even in same-donor oocytes, reinforcing the idea that the IVM environment may affect the amount of mRNA stored in the ooplasm. However, the oocytes matured *in vivo* included in our study were not obtained in natural conditions, since follicle growth and LH surge were stimulated with exogenous hormones. Thus, the difference observed in the present study between oocytes matured *in vivo* and *in vitro* might not fully represent all possible differences between oocytes derived from a single ovulation of a natural estrous cycle and oocytes submitted to *in vitro* maturation. We also found that the relative abundance of some transcripts may also differ when *in vitro* maturation is performed with oocytes obtained from pools with different donors.

*In vitro* maturation decreased the amount of polyadenylated transcripts of genes associated to maternal effects and to stress in both IVM groups (IN VITRO-OPU and IN VITRO-Abattoir), when compared to *in vivo* maturation (IN VIVO-OPU). Maternal-effect genes *Mater* and *Zar1* play an important role in the development of mouse oocytes after fertilization (Tong *et al*., 2000; Wu *et al*., 2003), while *Gdf9* is an oocyte-secreted factor present in oocyte-somatic cell interactions involved in oocyte developmental competence (Gilchrist *et al*., 2008). The lower abundance of transcripts encoding these genes seen in oocytes matured *in vitro* may be associated with decreased oocyte competence after IVM, when compared to oocytes submitted to *in vivo* maturation (van de Leemput *et al*., 1999; Humblot *et al*., 2005). Peroxiredoxins and HSPs are proteins involved in cell defense against oxidative stress (Martindale & Holbrook, 2002; Immenschuh & Baumgart-Vogt, 2005). *In vitro* culture conditions are known to increase the production of reactive oxygen species and thus cause oxidative damage to oocytes (Combelles *et al*., 2009; Morado *et al*., 2009). The lower abundance of *Prdx1* and *Hsp70.1* transcripts found after *in vitro* maturation can implicate in oocytes more sensitive to stressful conditions imposed by *in vitro* environment, which may contribute to the low developmental competence after fertilization.

The reasons behind the effects of *in vitro* maturation on the abundance of transcripts in the ooplasm have not been entirely elucidated, and may involve a combination of factors including a more stressful environment (Morado *et al*., 2009) requiring mRNAs for the synthesis of specific proteins, decreased ability of the oocyte transcription machinery (Bettegowda & Smith, 2007) to synthese news mRNAs, and degradation or deadenylation of transcripts during *in vitro* maturation (Thelie *et al*., 2009). A RNAseq study showed that some polyadenylated transcripts decreased in abundance during oocyte maturation, while others associated to cell-cycle progression, cytoskeletal organization, and macromolecule metabolism increased (Reyes *et al*., 2015). Despite these variations, polyadenylated mRNAs are relevant in meiotic resumption and further early embryo development (Piccioni *et al*., 2005; Brevini *et al*., 2007; Evsikov & Marín de Evsikova, 2009). Changes to polyadenylated transcripts may interfere with oocyte competence (Gandolfi & Gandolfi, 2001). However, the effects of *in vitro* maturation found in the present study may be specific for genes encoding high-demand proteins such as maternal-effect and antioxidant proteins, since we were unable to find differences in the abundance of transcripts encoded by genes related to apoptosis (*Bax* and *Bcl-2*) and chromatin unfolding (*Hmgn1*).

Interestingly, differences on relative abundance were found between the IN VITRO-OPU and IN VITRO-Abattoir groups, with lower amounts of *Prdx1, Hsp70.1, Gdf9*, and *Zar1* transcripts in the oocytes collected post-mortem at the abattoir. These oocytes also had lower amounts of the same transcripts when compared to the oocytes obtained from *in vivo* maturation followed by OPU (IN VIVO-OPU). Oocytes from the same donors were included in the IN VIVO-OPU and IN VITRO-OPU groups, while the IN VITRO-Abattoir group featured oocytes harvested from different cows. A possible reason for the low content of some specific transcripts in the oocytes collected post-mortem at the abattoir is the fact that these oocytes were harvested from donors with a different genetic background than the subjects included in the IN VIVO-OPU and IN VITRO-OPU groups. A recent study showed that cattle breed might affect oocyte mRNA abundance (Ticianelli *et al*., 2017). These findings highlight the need to compare maturation systems for efficiency using oocytes from the same donors in an attempt to avoid the misinterpretation of findings.

In general terms, this study showed that *in vitro* maturation might alter the abundance of key transcripts stored in the oocyte cytoplasm when compared to *in vivo* maturation induced by exogenous hormones, even in oocytes from the same donors. Therefore, IVM optimization is still required to improve molecular maturation regardless of oocyte origin.
